# Cultural Differences in Investing in Others and in the Future: Why Measuring Trust Is Not Enough

**DOI:** 10.1371/journal.pone.0040750

**Published:** 2012-07-24

**Authors:** Pascal Boyer, Pierre Lienard, Jing Xu

**Affiliations:** 1 Departments of Psychology and Anthropology, Washington University in St. Louis, St. Louis, Missouri, United States of America; 2 Department of Anthropology, University of Nevada, Las Vegas, Nevada, United States of America; 3 Department of Anthropology, Washington University in St. Louis, St. Louis, Missouri, United States of America; University of Utah, United States of America

## Abstract

Standard measures of generalized trust in others are often taken to provide reliable indicators of economic attitudes in different countries. Here we compared three highly distinct groups, in Kenya, China and the US, in terms of more specific attitudes, [a] people’s willingness to invest in the future, [b] their willingness to invest in others, and [c] their trust in institutions. Results suggest that these measures capture deep differences in economic attitudes that are not detected by standard measures of generalized trust.

## Introduction

Measures of generalized trust are often taken to provide reliable indicators of economic attitudes in different countries, capturing differences in the expectation that other economic agents are, on the whole, reliable and trustworthy [1]. This is part of a general focus on exogenous socio-cultural factors such as local norms and institutions as explanations of differential economic performance [2]–[4]. Economic models and empirical studies also emphasize the role of “social capital”, in the form of networks of trusted partners [5] that differ in extension and impact between cultures [6]–[9]. However, generalized trust is evaluated on the basis of highly general questionnaires, which makes the measure potentially misleading, aggregating different components of trust [10]–[12] and ignoring the issue of the radius of trust [1]–[13]. Generalized trust measures fail to distinguish between various targets, as people may e.g. trust members of their ethnic group and distrust all others; they also fail to indicate in which environments generalized trust would be the most rational attitude, in particular which institutions would be required [14].

We used a distinct set of finer-grained cross-cultural measures to elicit three crucial factors in economic behavior, namely [a] people’s time-discounting, or willingness to invest in the future, [b] their social discounting, or willingness to invest in others, and [c] generalized social trust (social capital) as well as trust in local institutions. We compared three sites with very different ecologies, social institutions and economies, located in China, the US and Kenya respectively (see Supplementary Materials S1 for details). Our Chinese participants are all urban dwellers in Yueyang (Hunan), ranging from students to employees. In Kenya, we recruited participants from the nomadic pastoralist Turkana tribe. The US participants were middle-class college students. These should maximize differences in attitudes, in the sense that they are in different ecologies and social systems. The Chinese participants have witnessed two decades of constant economic expansion and the dissolution of the former economic system. The Kenya herders where the research was conducted live in very harsh ecological, economic and socio-political conditions, practicing subsistence husbandry under constant threat of enemy raids, theft, drought and predators. Different ecological, economic and cultural conditions and socio-cultural institutions should differently affect the extent to which people trust others, and the extent to which they are confident in the future, as these depend on different aspects of the environment, social networks, and social norms for other-regarding, vs. distant forces (natural and political) for future-regarding preferences.

Standard instruments do not seem to support these expectations. The *World Value Survey* for instance (which does not include Kenya) suggests rather similar attitudes in China and the USA, as well as Uganda and Tanzania, two countries comparable to Kenya in population size and growth, ethnic patchwork, colonial and recent histories, wealth and infrastructure [15]. For items such as “trust in the government” and “trust in the civil services”, overall positive attitudes are less prevalent in the USA (38%, 42%) than China (93%, 86%), Uganda (78%, 69%) or Tanzania (83%, 71%). For trust in the “police”, the results are roughly similar in the USA (70%), Tanzania (67%) and China (80%). In brief, standard instruments suggest great similarities between the attitudes of people belonging to societies in strikingly different economic circumstance. The present studies aimed to provide a more sensitive probe into actual economic attitudes, specifically attitudes towards investment in others and in the future.

**Figure 1:**
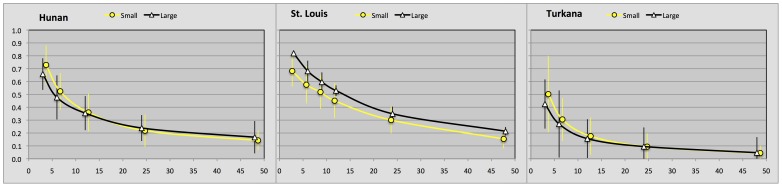
Discount curves for smaller and larger amounts of money as a function of time in months in three cultures.

## Methods

### Delay and Social Discounting

We used a discounting paradigm to gauge cross-cultural differences in future- and other-regarding preferences. Subjects were faced with choices, e.g. between $100 now and $200 a year from now, or between $100 for themselves and $200 for a sibling, with various delays and various target people (e.g. parent, acquaintance, stranger) respectively. Indifference points at each delay or for each type of person provided an estimate of a discount rate, that is, the utility for now of an amount *x* provided at delay *d*, or given away to person *x*. (See details in Supplementary Materials S1 and Figure S1).

**Table 1:** Summary of ANOVA results for the effects of amount and delay on discounting in each of the three sites, with effect-sizes.

	Factor	*F*	*df 1, 2*	*p*	*partial* η^2^
Hunan	Amount	1.62	1, 29	0.213	0.05
	Delay	207	5, 25	<.001	0.98
	Amount * Delay	2.12	5, 25	0.1	0.3
St. Louis	Amount	22.8	1, 24	<.001	0.49
	Delay	922	5, 20	<.001	0.99
	Delay * Amount	6.31	5, 20	<.001	0.61
Turkana	Amount	2.65	1, 43	0.11	0.06
	Delay	83	5, 39	<.001	0.91
	Delay * Amount	2.16	5, 39	0.08	0.22

**Table 2:** Omnibus ANOVA results for the effects of amount and delay on discounting in three sites, with effect-sizes.

Factor	*F*	*df*	*p*	*partial* η*^2^*
Culture	221	1, 96	<.001	0.96
Amount	10.82	2, 96	<.001	0.1
Delay	455	5, 92	<.001	0.96
*No significant interactions*				

Delay discounting is a familiar and highly reliable measure of future-regarding orientation [16]. Typically, most human subjects discount future rewards according to a hyperboloid function:
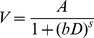
(1)where V is the present subjective value of the proposed delayed reward, A the amount promised, D the delay, and *b* and *s* are free parameters that stand for the general impatience factor and a scaling factor relating impatience to amount, respectively.

Social discounting is an extension of this protocol, taking social distance instead of time as providing the value of *D* in the above equation. Subjects are offered a choice between amount *x* for themselves and an amount *kx* for others, such as parent, sibling, friend, or perfect stranger. Jones & Rachlin showed that if people imagine others at various, numerically expressed degrees of social distance (e.g. between 1 [self] and 100 [perfect stranger]), their indifference points between *x* for self and *kx* for others also follows a hyperboloid curve, as a function of social distance [17]. In such discounting paradigms, imagined choices generally produce the same results as motivated choices with actual rewards [16].

**Figure 2:**
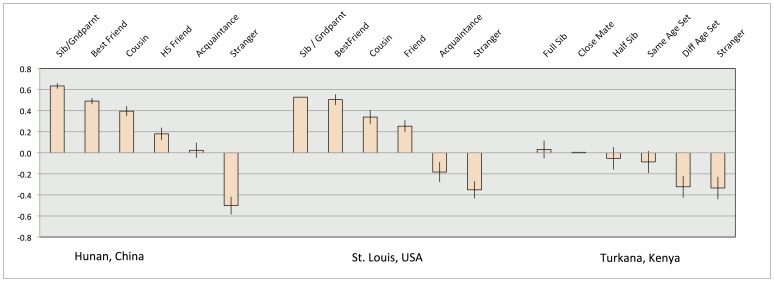
Social discount as a function of social positions in three different cultures.

### Cross-cultural Differences

In the domain of time-discounting, an extensive survey of students in 45 countries by Wang and colleagues showed that discount vary highly between sites [18], with two major findings. First, there were small or non-existent correlations between discounting and standard economic variables like income, inflation or current interest rates. Second, discount rates seemed to connect to “cultural” variables such as representations of time and the future. Additionally, this large-scale study showed that time-discounting was highly correlated with such measures as time-orientation and long-term orientation, so that the results of discount measures can serve as a proxy of a much broader attitude, a set of future-regarding preferences [18]. A limitation of this and other studies, however, was that participants in the non-Western sites were college students in economics or business.

**Figure 3:**
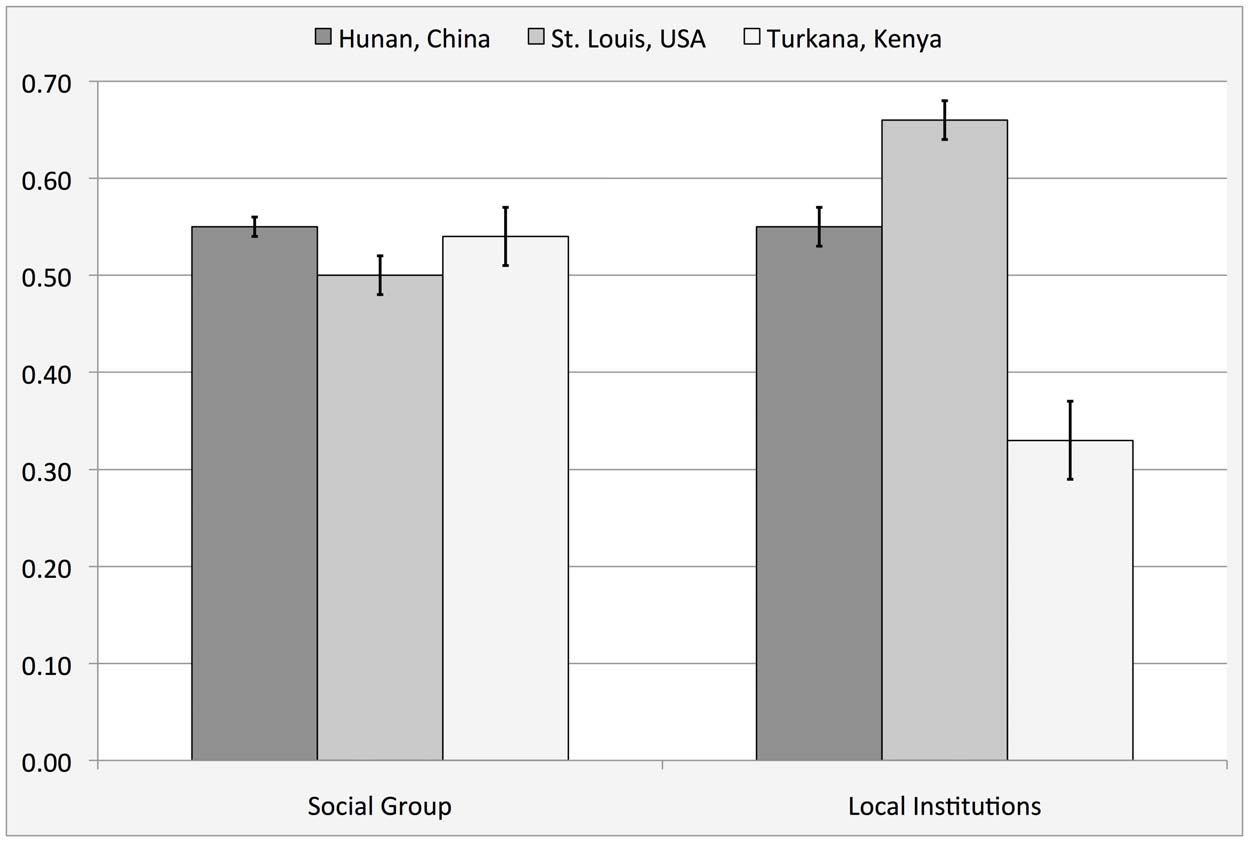
Trust in social group (an equivalent of social capital) and trust in local institutions in three cultures.

**Table 3:** Summary of ANOVA results for the effects of social distance on discounting in three sites.

	*F*	*df*	*p*	*partial* η*^2^*
Hunan	24.6	6, 36	<.001	0.804
St. Louis	11.9	5, 195	<.001	0.234
Turkana	4.6	5, 35	0.002	0.396

In the delay discounting situation, we offered participants choices that were calibrated to local conditions, e.g. Kenyan KES 2,000 provides approximately the same purchasing power as $100 in the US or RMB5 00 in China (when the research was conducted).

In the social discounting task, we used culturally realistic social positions. That is, instead of ranking abstract positions as in previous studies [19], we used local terms such as “sibling”, “best friend”, “high school friend”, etc. As a consequence the categories differ from one site to another, as some that make sense in some places (e.g. “high-school friend”) would be meaningless in others.

We ran a set of questionnaires to assess generalized social trust, as well as trust in local institutions. Our measure of social trust was not a single question, as in most surveys [20], but an aggregate of four to eight items concerning the likelihood that, in case of need, one could expect to receive help from neighbors, local associations, extended kin, etc. (see Supplementary Materials S1). Trust in institutions was similarly assessed with multiple items measuring confidence in police, justice, local and central officials, etc. (see Supplementary Materials S1).

We obtained written consent from all Chinese and US participants, and verbal consent from non-literate Kenyan participants, in accordance with IRB-approved protocols.

## Results

This report presents separate descriptives and tests for the different protocols. There was insufficient overlap in participants between all three protocols to allow for individual level correlations.

### Delay Discounting

To compute the discount rate, we first estimated an indifference point by taking the mean of smallest allocations to future and greatest allocations to present, at each delay. The values reported below are future gain/present gain ratios. For instance, preference for $1,000 a year from now over $800 now would imply a current utility >.8 for a delay of one year. Figure 1 summarizes the results for our three sites (more detail in Supplementary Materials S1).

The discount curves suggest different discount rates, where Kenyans are the steepest discounters, followed by the Chinese and then the US participants. In contrast to Du et al. [21] but in accordance with Wang et al. [18] we found a significant difference between US and Chinese participants. In accordance with Wang et al. [18] African participants showed the steepest discounting rate. Detailed analyses for the different sites show that there were effects of delay in all sites, and a small effect of amount in the USA, but not in China or Africa, see Table 1. Differences between sites are confirmed by an omnibus ANOVA, as indicated on Table 2.

### Social Discounting

At each trial, participants had to choose between money for themselves and some money for others, to be called $_Me_ and $_Other_. So for instance participants have to choose between $100 for Me and $200 for Other. To summarize participants’ choices, we computed a “social utility” (SU) index, supposed to gauge the utility for each participant of allocating money to other individuals, depending on social distance (see Supplementary Materials S1). These raw values are scaled to the [−1,1] interval and displayed in Figure 2.

Results suggest a clear difference between the USA and China on the one hand, where positive and negative sharing is distributed along different positions, and Kenya where there is much less sharing with any social positions. To compare the social discount choices between sites, we added the SU for each subject across social positions, giving us a broad measure of how much participants would be willing to sacrifice resources for all others combined, regardless of social distance. We then compared the results obtained in different sites. A one-way ANOVA shows a significant effect of site, F(2,105) = 12.2, *p*<.001. Planned comparisons (independent sample t-tests) show a significant difference between Kenya and the two other sites, both *p*s<.001, but no significant difference between China and USA, *p* = .54.

### Generalized Social Trust and Trust in Local Institutions

Averages for the aggregate measures of generalized social trust (trust in one’s social group) and trust in local institutions are summarized in Figure 3. Contrary to expectations we did not find a difference in social trust, which may be explained by the fact that our instrument measured people’s concrete expectations of help from actual people, other than a general statement to the effect that “people are trustworthy”. We did find an important difference in trust in local institutions, as Kenyan participants have much less trust in their local officials or bureaucrats than US participants. An omnibus ANOVA showed no effect of culture on the measure of social trust, F_(2,326)_ = 1.15, *p* = .32; and a significant effect of culture on trust in local institutions, F_(2,326)_ = 28, *p*<.001, *partial* η^2^ = .15. Results of specific ANOVAs for each site are summarized in Table 3.

## Discussion

These studies show deep cultural differences in economic attitudes that are not captured by measures of generalized trust and social capital. Delay discounting results are consistent with previous evidence of very steep discounting in Africa and fairly steep rates in China, compared to the USA [18], [21]. The results also show clear differences in social discounting. There is however a clear dissociation between future- and other-regarding attitudes. Chinese participants show both high other-regarding preferences (like US, unlike Kenya) and low future-regarding preferences (like Kenya, unlike US). This provides an empirical confirmation to the suspicion that generalized trust is at best a poor measure of economic attitudes [10].

The origin of these differences cannot be found in estimates of how much help people can expect from known others, a precise measure of “social capital”, which were similar in our three sites despite enormous economic and institutional differences. Cultural differences are more consistent with different histories and ecologies. Turkana pastors live in a high-uncertainty environment, including recurrent droughts and raids from enemy tribes, as well as intra-tribal rivalries (*22, 23*). Our Chinese participants have experienced massive economic growth and market integration, with a widespread perception that local institutions cannot be trusted [24]–[25].

Rather than generalized trust, economic development requires both an expectation that the future will be stable (expressed here as shallow time-discounting), a willingness to create networks of reciprocal obligations (expressed here in shallow social discounting), and reliable institutions (expressed here as trust in local representatives of these institutions). All three factors display important cultural differences. Policy-makers, elected officials, corporations and other institutional designers should measure potential obstacles to development, not on the basis of vague perceptions of trust or social capital, but on more precise tools revealing fundamental attitudes to the future and to others.

## Supporting Information

Figure S1 **Theoretical discount curves for different values of the discount factor k used to generate the five different amounts offered at each specific delay in the delay-discounting task.**
(TIF)

Supplementary Materials S1 (DOCX)
