# Supplementary figures and images for: Cultural Differences in Investing in Others and in the Future: Why Measuring Trust Is Not Enough
Source: PLoS One. 2012 Jul 24;7(7):e40750. doi: 10.1371/journal.pone.0040750 (PMC3404099; doi:10.1371/journal.pone.0040750)

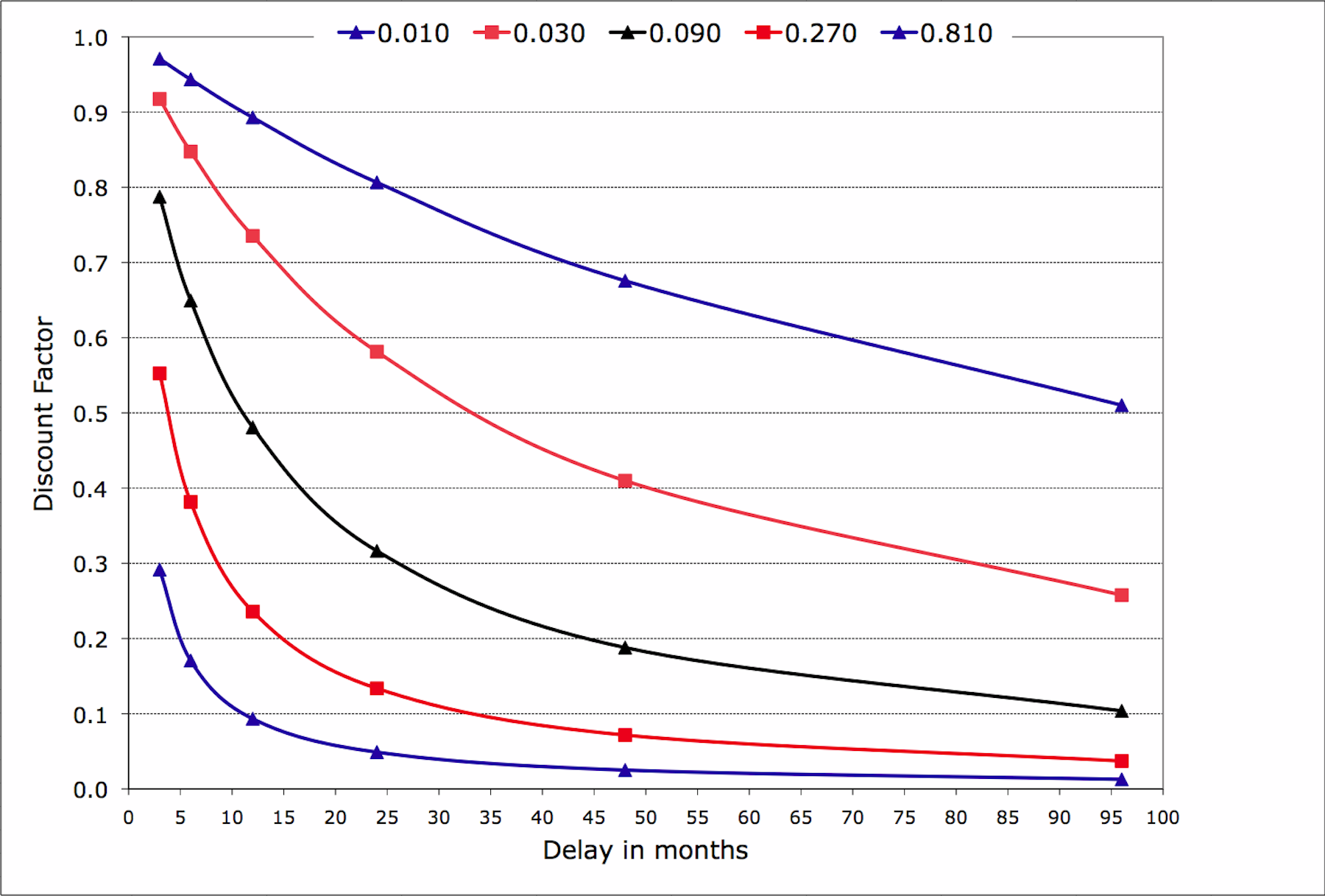

Supplement: Figure S1 — Theoretical discount curves for different values of the discount factor k used to generate the five different amounts offered at each specific delay in the delay-discounting task. (TIF) [file pone.0040750.s001.tif]
