# Supplementary material for: Cultural Differences in Investing in Others and in the Future: Why Measuring Trust Is Not Enough
Source: PLoS One. 2012 Jul 24;7(7):e40750. doi: 10.1371/journal.pone.0040750 (PMC3404099; doi:10.1371/journal.pone.0040750)
Supplement: Supplementary Materials S1 — (DOCX) [file pone.0040750.s002.docx]

Supplementary Materials to:

Cultural Differences in Investing In Others And In The Future:
Why Measuring Trust Is Not Enough

These supplementary materials comprise three sections:

[1] Information on participants;

[2] Information on experimental methods;

[3] Data analysis.

# Further information on participants

## China

[1] Discounting Studies. A total of 36 subjects from Yueyang, China participated in this experiment. During the delay-discounting test, because of technical problems, only the results of 32 participants (21 female) were available for analysis, ages ranging from 16 to 55, (*M*=33.2, *SD*=15). Generation was considered an important variable under the Chinese social context of family-planning policy, we called subjects as “older” or “younger” generation with a cut of date of both at 1981 (28 year-old at the time of experiment).

[2] Trust questionnaires. A total of 229 participants (110 women) from Yueyang, China, ages ranging from 16 to 18 (*M*=16.8, *SD*=0.6).

## United States

[1] Discounting Studies. Because of the abundance of discount data for US populations, this small sample is only used as confirmation that the protocols used here provide results consistent with slightly different methods. (See comments on country comparisons in main text and here below). Twenty-five undergraduate students (13 women) at Washington University participated against course credit. Ages ranged from 18 to 25, *M*=20.

[2] Trust questionnaires. Seventy-nine students (53 women) at Washington University participated against course credit, age ranging from 18 to 30 (*M*=20).

## Kenya

[1] Discounting Studies. All 44 participants (22 each gender, ages ranging from 18 to 78, *M*=34.8, *SD*=13.1) were recruited among the *Ngilukumong* territorial section of the Turkana nomadic pastoralists of Kenya, in the area of Kalobeyei, Turkana West district. They took part in the study against a KES 200 compensation. All subjects were illiterate, and all of them were engaged in nomadic animal husbandry as their primary source of income.

[2] Trust questionnaires. All twenty-four participants (8 women, ages ranging from 25 to 60. *M*=39, *SD*=10) were recruited among the *Ngilukumong* territorial section of the Turkana nomadic pastoralist of Kenya, in the area of Kalobeyei, Turkana West district. They took part in the study against a KES 200 compensation. All subjects were illiterate, and all of them were engaged in nomadic animal husbandry as their primary source of income.

# Further information on methods

## Delay Discounting

*Materials and list of choices.* The delay discounting task consisted in a series of consecutive, randomly ordered choices between a sum of money now and a larger sum of money at a specified date in the future, e.g. six months from now. The choices concerned a small sum, $100 in the US trials, and the local equivalent in purchasing power in both China and Kenya. 6 levels of delay were presented in each amount level: 1 month, 3 months, 6 months, 1 year (12 months), 2 years (24 months), 4 years (48 months).

To create the 60 trial values, we generated several sets of discounted values for each of the two amounts and the different delays. Specifically, we generated five theoretical discount curves, with different values of *k*. These were chosen so as to comprise values above and below the discount rates observed in the literature (Du, et al., 2002; Green & Myerson, 2004; Green, Myerson, & McFadden, 1997). Based on these findings, choosing *k* between .005 and .567 should be enough to cover most choices, including extreme ‘discounters’ and ‘non-discounters’. Five discount rates were thus chosen. The 60 trials were generated through combination of amount level, delay level and assumed discount rates (6*2*5=60 trials), see Figure S1 in the Supporting Information.

*Procedure.* All participants were tested individually, in their native languages, in quiet rooms. Participants were informed of the anonymous nature of this experiment. In China and the US, the participants followed instructions on a laptop computer, entering their NOW or LATER choice at each trial. In Kenya, choices appearing on the computer screen were read out by the experimenter’s research assistant who then entered the response. The delay-discounting instructions read as follows (with appropriate translations for China and Kenya):

In this study, we will ask you to make several choices between getting some money NOW or getting some amount of money in some time. For instance, the computer will ask you: Which would you prefer, 100 now or 150 in three months?

The choices are imaginary, but please try to answer as you would if you were faced with the real choice. For each new choice, think of which sounds better for you, and click the sum of money in the box. To start, please click the mouse.

When the participant was reading the instructions, the experimenter emphasized that the amount of money in future was guaranteed to be ready for the participant on time.

## Social Discounting

*Materials and list of choices*. In the social discounting test, a constant amount for others (RMB 500), and 8 different amounts for self (60, 90, 140, 210, 325, 500, 770, and 1200) were used. The factor/ratio of 0.65 is used as the criteria under which the 8 amounts were generated (325=500*0.65, 770=500/0.65, 210=325*0.65, etc.).

The different social positions could not be the same in the different sites. First, an apparently similar kinship position (e.g. “cousin”) denotes vastly different assumptions, e.g. about commonality of interest, in different cultures. Second, some social positions of great importance for solidarity and exchange only exist in particular cultures, such as “member of the same age-set” in some East-African tribes or “compadre” in Hispanic cultures. So we used a set of social positions, which, on the basis of previous anthropological work, would correspond to salient points in each culture in terms of typical exchange relations. The positions were the following:

China: Parents, Sibling or grandparent, Cousin, Best friend, High-school friend, Acquaintance, and Stranger. Because the younger subjects in this experiment are from single-child families, while the older subjects were not, the category B would mostly denote “grandparent” to the younger subjects and “sibling” to the older ones.

USA: Parents, Sibling or grandparent, Cousin, Best friend, Cousin, Friend, Acquaintance, and Stranger.

Kenya: Full sibling (same mother and father), Half sibling (same father), Close mate, Age-mate member of the same age-set, Age-mate member of a different age-set, Acquaintance, and Stranger. The difference between half- and full siblings is relevant as these social relations, in a polygynous household, entail very different levels of solidarity and cooperation. Formally defined age-sets are a crucial institution among the Turkana. Each person belongs to an age-set and engages in important economic and political activities with members of the same set, among whom there is an expectation of higher solidarity and cooperation than across sets.

In both China and Kenya, the translation conveyed the meaning that an “acquaintance” is personally known but the relation has no special emotional tenor, and a stranger is someone who was not previously encountered.

*Procedure*. In China and the US, participants were seated in front of a laptop computer and could read instructions and input their responses on the keyboard. In Kenya, the instructions were read out by the research assistant, followed by a short comprehension test. The English version of the social-discounting instructions read as follows:

In this study, we will ask you to make several choices between getting some money for YOURSELF or getting some amount of money for OTHERS. For instance, the computer will ask you: Which would you prefer, 200 for yourself or 320 for one of your cousins?

The choices are imaginary, but please try to answer as you would if you were faced with the real choice. For each new choice, think of which sounds better for you, and click the sum of money in the box.

To start, please click the mouse.

In China and Kenya these were translated into the vernacular, and all testing took place in the participants’ native language.

## General trust and trust in institutions questionnaires

The social trust questionnaire comprised two parts. One was a single item similar the standard World Value question, but spread over several items. Questionnaire requested that people evaluate their agreement on a 4 point scale with the statements: “(a) Most people in this village/neighborhood are basically honest and can be trusted; (b) People are always interested only in their own welfare; (c) Members in this village/neighborhood are always more trustworthy than others; (d) In this village/neighborhood one has to be alert or someone is likely to take advantage of you; (e) If I have a problem there is always someone to help you; (f) I do not pay attention to the opinions of others in the village/neighborhood; (g) Most people in this village/neighborhood are willing to help if you need it; (h) This village/neighborhood has prospered in the last five years; (i) I feel accepted as a member of this village/neighborhood; (j) If you drop your purse or wallet in the neighborhood, someone will see it and return it to you.” The second part comprised a single item, as follows: “Suppose your neighbor suffered an economic loss, e.g. (RURAL: “animal death”; URBAN “job loss”). In that situation, who do you think may assist him/her financially? [Record first three mentioned.] 1. No one would help; 2. Family; 3. Neighbors; 4. Friends; 5. Religious leader or group; 6. Community leader; 7. Business leader; 8. Police ; 9. Family; 10. Patron/employer/benefactor; 11. Political leader; 12. Mutual support group to which s/he belongs; 13. Assistance group to which s/he belongs; 14. Other; 15. Don’t know/not sure; 16. No answer” The specific list was modified for each culture, eliminating concepts that would not be culturally familiar (e.g. mutual assistance groups in the US or neighbours in Turkana).

The institutional social trust questionnaire included the following items:

How much confidence (from 1 to 4) do you have in the following institution: legal system, police, local administration, central government? Do you think any of the following could be a threat to you or your family: legal system, police, administration, government?

The phrasing of the items was adapted to the different cultures.

# Data analysis

## Delay discounting

We asked for subjects’ responses in randomly presented binary choices between some money now and a larger amount later. This protocol does not automatically provide a value for the switching point at which subjects start to prefer a delayed reward to the immediate one. To extrapolate the switching point, we computed the average of [a] the minimal delayed-value at which the subject preferred to wait, and [b] the maximum value at which the subject preferred to take the reward now. These extrapolated values were then converted into a ratio of the nominal value to compute the discounting factor. For instance, a switching point of 750 at six months, against an immediate reward of 500, would imply that the discount rate at six months for 500 is equal to 500/750=.667.

## Social discounting

In the protocol used here, we cannot plot discount choices over social distance using the latter as a continuous or ranked variable as done in other studies (Rachlin & Jones, 2008), because we cannot *a priori* evaluate the subjective distance participants establish between themselves and different social positions, in contrast to the time dimension in delay discounting. Also, it is difficult to establish a “switching-point” where people reverse choices, e.g. switching from selfish to generous choices. In many cases people make apparently inconsistent choices, e.g. sacrificing $40 to give their cousin $100 but refusing to sacrifice $25 for the same outcome. So we need a measure of people’s choices that is not dependent on such a consistent mid-point yet reflects the variable “generosity” of choices concerning different social positions.

We computed the “social utility” SU index as follows:

- For “give to OTHER” decisions, SU = $Me / $Other
- For “keep for ME” decisions, SU = –($Other / $Me )

The rationale is that, for “give to OTHER” decisions, SU represents the amount the participant is willing to sacrifice to give money to others. So the more money is forgone (larger numerator), the more ‘generous’ the participant is. Participants who only choose OTHER when $Other is very large (e.g., either $100 for me or $5,000 for other) get a lower SU score as the denominator is large. For “keep for ME” decisions, SU represents as a negative value the amount the decision costs to Other. So the more money a participants Other by choosing ME, the higher the absolute value of this ratio, and therefore the lower the SU (as it is negative). Participants who choose “keep for ME” even when $Me is very low (e.g., either $10 for me or $500 for other) get a greater absolute value of SU as the denominator is small, and that value is signed as negative to reflect the “selfish” nature of these choices.

These raw values are then scaled to the [-1,1] interval by linear transform, to adjust the score to the decisions that were actually available to the subjects, and which differ slightly from site to site.

To compare SUs between sites, we cannot directly compare participants’ SU towards a particular social position, as we did not use the same list of positions in different sites. A rough but relevant measure is provided by adding the SUs for all social positions for each participant. This gives us a measure of how much they are prepared to sacrifice to other people’s benefit, regardless of what social positions they occupy. This is relevant to the present discussion, in the sense that a society where this average SU is higher is one where people can expect others to be more sensitive to their own needs.

## Trust questionnaires

The measure of general social trust is an aggregate of participants’ responses to items concerning help in case of misfortune, excluding the family and parents, with reverse coding where appropriate. This was then scaled [0,1].

The measure of institutional trust is an aggregate of participants’ responses to the items concerning trustworthiness of and possible danger from police, justice system and local officials, with reverse coding where appropriate. This was then scaled [0,1].
